# Occupational physical activity and resting blood pressure in male construction workers

**DOI:** 10.1007/s00420-023-02006-2

**Published:** 2023-09-19

**Authors:** Jerry Öhlin, Per Liv, Martin Andersson, Bengt Järvholm, Lisbeth Slunga Järvholm, Albin Stjernbrandt, Viktoria Wahlström

**Affiliations:** Section of Sustainable Health, Department of Public Health and Clinical Medicine, Umeå University, 901 87 Umeå, Sweden

**Keywords:** Systolic blood pressure, Diastolic blood pressure, Occupational physical activity, Construction workers, Cardiovascular health

## Abstract

**Objective:**

This study investigated the association between occupational physical activity (OPA) and resting blood pressure in a cohort of Swedish construction workers.

**Methods:**

The final sample included 241,176 male construction workers. Occupations with low OPA were foremen and white-collar workers. The most frequent occupations in the medium OPA group were electricians, pipe workers, and machine operators, and in the high OPA group woodworkers, concrete workers, and painters.

**Results:**

Mixed effects models showed higher systolic and lower diastolic blood pressure with higher OPA, but the associations varied depending on the year of participation and participant age as shown by significant interaction terms (OPA*age, OPA*calendar year, age*calendar year). Age-stratified linear regression analyses showed a pattern of slightly higher systolic (1.49, 95% confidence interval: 1.08–1.90 mmHg) and lower diastolic (0.89, 95% confidence interval: 0.65–1.13 mmHg) blood pressure when comparing low with high OPA, but not among the oldest age groups.

**Conclusion:**

Despite a rather large contrast in OPA, the differences in systolic and diastolic blood pressure according to OPA were small.

## Introduction

Hypertension is the primary risk factor for cardiovascular disease (Fuchs and Whelton 2020) which in turn is the leading cause of death worldwide (Roth et al. 2020). Physical activity (PA) is a recommended non-pharmacological treatment for hypertension (Whelton et al. 2018). Although intensity of both occupational PA (OPA) and leisure-time PA (LTPA) are commonly measured via heart rate, the physiological responses might differ (Holtermann et al. 2018). OPA may have detrimental health effects, as studies have found higher OPA to be associated with poor self-rated health (Van den Berge et al. 2022), long-term sick-leave (Gupta et al. 2020), disability benefits (Järvholm et al. 2014) and higher mortality among men (Coenen et al. 2018). Further suggested reasons for this PA paradox are that many occupations with high OPA require workers to be physically active for the majority of waking hours during workdays, which results in insufficient time for recovery. Further reasons include insufficient recovery from exertion, increased systemic inflammation, and elevated 24-h blood pressure (BP). OPA may cause sustained high blood pressure that persists outside work (Holtermann et al. 2018) which have been suggested as a plausible physiological contributor to the increased risk of cardiovascular disease and mortality among workers with high OPA (Clays et al. 2012).

A study used cardiac load measured via ambulatory 24-h heart rate to estimate high OPA and found it was associated with increased resting diastolic BP among participants under 47 years old (Korshøj et al. 2019). The picture is not entirely clear, however, as a study found that moderate OPA was associated with lower risk of new-onset hypertension among Chinese men and women (Li et al. 2021), and high OPA was associated with greater longevity in a study of Norwegian men (Dalene et al. 2021). Also, a recent review of the association between OPA and cardiovascular disease mortality stated that residual confounding due to socioeconomic status cannot be ruled out (Cillekens et al. 2022). Thus, further studies on the relationship between OPA and BP are warranted utilizing cohorts with similar socioeconomic status, especially in higher ages and in professions with high OPA such as construction workers.

Our study aimed to investigate if OPA is associated with resting BP in male construction workers. Our hypotheses were that higher OPA is associated with higher systolic and higher diastolic BP at rest and that these associations are more pronounced in higher age.

## Method

### Design

Our study compared differences in BP between levels of OPA in a cohort of male construction workers from 1971 to 1993.

### Sample

This study is based on a cohort of construction workers who participated in health surveys in a nationwide occupational health service (Bygghälsan) between 1971 and 1993 (Jackson et al. 2019). Women were excluded from this study as they only constituted 5% of the cohort and mostly had occupations for which work environmental assessment had not been performed, and hence, no OPA measure was available. Men with body mass index (BMI) between 18.5 and 35 kg/m^2^, with at least one survey participation and data on age, smoking, occupation, and systolic and diastolic BP measurements were included. From our sample, 37,731 men were excluded due to outside age range (20–69 years), 7755 outside BMI range (≥ 18.5 to < 35 kg/m^2^), 553 with very high or very low BP measures (systolic > 240 or < 80, diastolic > 130 or < 50), and 82,499 with missing occupational category code or occupational category code without heart rate measurement. A full list of the occupational categories are available in Appendix 1, and further details are available in a previous study (Jackson et al. 2019).

### Covariates

Age, weight, height, occupational category, and smoking (never, former, current, or unknown) were retrieved from participants' most recent health surveys, which were performed by occupational health nurses. BMI was calculated as weight in kg divided by height in meters squared and categorized according to World Health Organization’s criteria (Weir and Jan 2022), normal (≥ 18.5 to < 25), pre-obese (≥ 25 to < 30), or obese class 1 (≥ 30 to < 35).

### Occupational physical activity

All participants in Bygghälsan were asked to report their job titles according to the Swedish construction industry work codes at the time of participation (212 job titles before 1986 and 90 job titles from 1986 onward). Work assessment profiles rating relevant exposures (e.g., pollutants, vibration, injury risk, musculoskeletal load, and heart rate) were developed based on observations and measurements on 5–15 individuals from each of the 187 job titles. These job titles were grouped into 22 occupational categories by technical experts in the industry according to similar work tasks and training. Heart rate was measured with a long-term electrocardiogram recorder (Cardirec HRI-3), excluding individuals with known cardiovascular disease. The peak and average heart rate was grouped into five categories (1: < 75, 2: 75–100, 3: 101–125, 4: 126–150, and 5: > 150 beats per minute). This study used the average heart rate as a measure of OPA. Heart rate was not measured in white-collar workers or foremen, and for this study, they were categorized into the low OPA group. The remaining occupational groups had a mean heart rate between 75–100 or 101–125 and categorized as medium and high OPA, respectively.

### Blood pressure

At each health survey, resting systolic and diastolic BP was measured to the nearest 2 mmHg on the brachial artery with a stethoscope and standard mercury sphygmomanometer on the upper arm in a supine position after five minutes of rest.

### Statistical analysis

Continuous variables are presented as mean ± standard deviation, ordinal and nominal variables as counts (percentage). The longitudinal association between OPA (modeled as a categorical variable) and systolic and diastolic BP from 1972 to 1993 was investigated using mixed effects models. The models included OPA, BMI, smoking habits, calendar year and age, and interactions between OPA and age, OPA and calendar year, and age and calendar year. A random effect for participant was also included to account for repeated participations, while all other effects were fixed. Continuous independent variables were entered into the model using restricted cubic splines with three knots to account for non-linear effects. These mixed effects models were used to visualize the effects in line plots, where predicted values of BP as functions of OPA and age were estimated along with 95% confidence intervals, adjusted at calendar years 1972, 1982, and 1992 to demonstrate the effect of calendar years. The associations between OPA and BP were also investigated with linear regressions in ten-year age groups from 20–29 to 60–69, adjusting for age (with restricted cubic splines with three knots to account for non-linear effects), BMI category, and smoking habits. Average resting systolic and diastolic BP from participants health surveys (median 3 and range 1–13) were used in the linear regressions. Confidence intervals not containing zero were considered statistically significant. No issues of multi-collinearity appeared in the models. All statistical analyses were performed in R Statistical Software (V 4.2.2; R Core Team 2022) with mixed effects models estimated with lmer from the lmer package.

### Ethical approval

This study was approved by the ethical review board in Stockholm, Sweden (Dnr 2020-01927), and performed according to the Declaration of Helsinki.

## Results

A total of 241,176 males were included in the analyses. Their mean age was 42 ± 13 years (Table 1). Sixty-three percent were normal weight (BMI ≥ 18.5 to < 25 kg/m^2^), 32% pre-obese (BMI ≥ 25 to < 30 kg/m^2^), and 4.3% obese class 1 (BMI ≥ 30 to < 35 kg/m^2^). Thirty four percent were never smokers, 21% former and 33% current smokers. The majority, 59%, had high OPA, 14% low, and 27% medium. Occupations constituting the low OPA group were foremen, *n* = 24,545 (73%) and white-collar workers, *n* = 9 286 (27%). The most frequent occupations in the medium OPA group were electricians, *n* = 24,610 (37%), plumbers and pipe fitters, *n* = 19,935 (30%), and heavy machinery operators, *n* = 8394 (13%). The most frequent occupations in the high OPA group were woodworkers, *n* = 52,792 (37%), concrete workers, *n* = 32,278 (23%), and painters, *n* = 19,368 (14%). For a complete list of occupations according to OPA level, see Appendix 1.

**Table 1:** Characteristics of study participants

Characteristic	Total	Occupational physical activity		
		Low, *N* = 33,831	Medium, *N* = 65,654	High, *N* = 141,691
Age	42 ± 13	44 ± 12	39 ± 12	43 ± 13
Smoking status				
Never	81,097 (34%)	12,350 (37%)	22,154 (34%)	46,593 (33%)
Former	49,482 (21%)	7036 (21%)	13,457 (20%)	28,989 (20%)
Current	80,359 (33%)	9424 (28%)	21,531 (33%)	49,404 (35%)
Unknown	30,238 (13%)	5021 (15%)	8512 (13%)	16,705 (12%)
BMI category				
Normal weight	153,118 (63%)	20,582 (61%)	42,018 (64%)	90,518 (64%)
Pre-obese	77,668 (32%)	11,729 (35%)	20,660 (31%)	45,279 (32%)
Obese CL 1	10,390 (4%)	1520 (4%)	2976 (5%)	5894 (4%)

Data presented as mean ± standard deviation or *n* (percentage, rounded to nearest integer). BMI (kg/m^2^): normal (≥ 18.5 to < 25), pre-obese (≥ 25 to < 30), or obese CL 1 (≥ 30 to < 35)

**Fig. 1:**
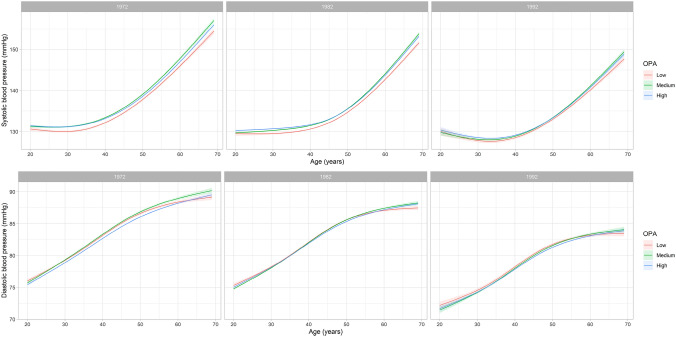
Predicted values of systolic (upper row) and diastolic (lower row) BP as function of OPA and age with 95% confidence interval

The mixed effects models included 241,176 individuals, with a total of 857,075 survey participations (180,676 workers participated more than once, and median number of participations was 3). The mixed effects models showed higher systolic and lower diastolic BP with higher OPA. However, the associations varied depending on the year of participation and participants age shown by significant interaction terms between OPA and age, OPA and calendar year, and age and calendar year. Figure 1 shows the predicted values of systolic BP (upper panels) and diastolic BP (lower panels) as a function of OPA and age in 1972, 1982, and 1992.

Table 2 presents results from the linear regressions, showing the difference in systolic and diastolic BP in mmHg between OPA levels (low minus medium, low minus high, and medium minus high) with corresponding 95% confidence interval, stratified by age groups. Participants with low OPA had lower systolic BP compared to participants with medium OPA, although not a significant difference in the youngest and oldest age groups. In all age groups, participants with low OPA had lower systolic BP compared to participants with high OPA. In the youngest age group, participants with medium OPA had a significantly lower systolic BP than those with high OPA. In contrast to systolic BP, lower OPA was associated with higher diastolic BP, but only among a few of the younger age groups (Table 2). The unadjusted age-stratified analyses of systolic and diastolic BP showed similar results as the adjusted (Appendices 2 and 3).

**Table 2:** Differences in systolic and diastolic blood pressure (in mmHg) between occupational physical activity (OPA) groups stratified by age

Age groups	Low–medium OPA	Low–high OPA	Medium–high OPA
Systolic blood pressure			
20–29	− 0.20 (− 0.53, 0.13)	**− 0.50 (− 0.82, − 0.19)**	**− 0.30 (− 0.50, − 0.11)**
30–39	**− 0.59 (− 0.86, − 0.31)**	**− 0.75 (− 1.00, − 0.49)**	− 0.16 (− 0.36, 0.03)
40–49	**− 0.85 (− 1.18, − 0.52)**	**− 0.71 (− 1.01, − 0.40)**	0.14 (− 0.11, 0.40)
50–59	**− 1.25 (− 1.72, − 0.78)**	**− 1.49 (− 1.90, − 1.08)**	− 0.25 (− 0.60, 0.10)
60–69	− 0.64 (− 1.40, 0.12)	**− 1.05 (− 1.64, − 0.45)**	− 0.40 (− 0.99, 0.17)
Diastolic blood pressure			
20–29	**0.88 (0.63, 1.13)**	**0.89 (0.65, 1.13)**	0.01 (− 0.13, 0.16)
30–39	**0.28 (0.08, 0.48)**	**0.47 (0.28, 0.65)**	**0.19 (0.05, 0.33)**
40–49	0.06 (− 0.16, 0.28)	**0.39 (0.19, 0.59)**	**0.33 (0.16, 0.50)**
50–59	− 0.05 (− 0.31, 0.21)	− 0.07 (− 0.30, 0.16)	− 0.02 (− 0.21, 0.18)
60–69	0.01 (− 0.38, 0.41)	− 0.25 (− 0.56, 0.06)	− 0.26 (− 0.56, 0.04)

Difference in mmHg (95% confidence interval). Adjusted for BMI category, smoking status, and age. Numbers in bold indicate statistically significant differences, i.e. confidence intervals not containing zero

## Discussion

We hypothesized that higher OPA would be associated with higher systolic and diastolic BP and that these associations would be more pronounced in older workers. Higher OPA was associated with higher systolic BP as expected, but the effect was minor and mainly seen among younger workers and not older workers, contrary to our hypothesis. Higher OPA was also associated with lower systolic BP mostly among younger workers, contrary to our hypothesis.

In line with our results, previous studies have shown high self-reported OPA to be associated with higher resting and ambulatory systolic BP (Barengo et al. 2006; Clays et al. 2012). However, the study by Clays et al. did not find an association between device-measured OPA and ambulatory systolic BP. As they measured OPA with hip-worn accelerometers, the total PA may have been underestimated. This is due to many manual occupations entail static and flexed position of the back, arm movements, and heavy manual handling, the latter having been shown to potentially increase hypertension risk among people over 50 years of age (Korshøj et al. 2020).

The association between higher OPA and higher systolic BP could be due to socioeconomic factors (Sun et al. 2022). In the youngest age group, we found higher systolic BP associated with higher OPA also when comparing medium with high OPA, which include occupations with very similar socioeconomic status. Furthermore, almost all workers in the construction industry in Sweden attend profession-specific schools and few change career during their lifetime. Therefore, it is unlikely that socioeconomic status is the main determinant of the differences in systolic BP.

Contrary to our hypothesis, we found that higher OPA was associated with lower, not higher, diastolic BP among participants below 50 years of age, which have also been found among Finish men aged 25 to 64 years (Barengo et al. 2006). Higher OPA measured with technical measurements of relative heart rate reserve was found to associate with higher diastolic BP among older workers (> 47 years of age) but not younger workers (Korshøj et al. 2019). The lack of an association between OPA and diastolic BP in higher ages may be due to age-related cardiovascular changes. Diastolic BP increases with age due to rising arterioral resistance, but then declines due to stiffening of the large arteries (Singh et al. 2022). However, the decline in diastolic BP usually manifests around 60 years of age (Cheng et al. 2012) and is therefore not a probable explanation for the lack of an association between OPA and diastolic BP from 50 to 69 years of age in our sample.

Our study could not measure physical activity outside of the occupational setting, which is a limitation. Moreover, we could not measure relative heart rate, which would be a more precise measure of OPA. Another limitation is the lack of adjustment for cardiovascular disease, antihypertensive treatment, and socioeconomic status. However, antihypertensive treatment adjustments have been found to have little impact on the differences in BP between socioeconomic groups (Bann et al. 2020), while we adjusted for BMI as it has been found to attenuate the association between socioeconomic status and BP (Bann et al. 2020). Strengths of this study entail a large sample of a homogenous group of construction workers, measures of BP by occupational health nurses, and statistical adjustments for time trends in BP.

In this sample of construction workers, the average systolic and diastolic BP seemed to decline from 1972 to 1993. Declining systolic BP has also been found in the Northern Sweden MONICA project (Eriksson et al. 2017), and possible explanations for the decreased cardiovascular disease risk entail dietary changes, improved antihypertensive treatment and lower rate of smoking (Eriksson et al. 2011), which tentatively could also be the case for the construction workers in our study.

Future studies should investigate how longitudinal changes from repeated measures of OPA influence systolic and diastolic BP, the possible influence of sex and age, and consider possible non-linear effects. In conclusion, our study found higher systolic and lower diastolic BP with increasing OPA in male construction workers, but mainly among the younger participants. Furthermore, these differences were of low clinical significance, despite occupations ranging from heavy OPA such as woodworkers, concrete workers and painters, to white-collar workers and foremen with low OPA.

## Data Availability

The datasets generated during the current study are available from the corresponding author on reasonable request.

## Appendix 1. Distribution of occupations according to occupational physical activity level

**Table:**

Occupation	Low OPA, *N* = 33,831	Medium OPA, *N* = 65,654	High OPA, *N* = 141,691
Foremen	24,545 (73%)		
White-collar workers	9286 (27%)		
Electricians		24,610 (37%)	
Plumbers and pipe fitters		19,935 (30%)	
Heavy machinery operators		8394 (13%)	
Drivers		3928 (6.0%)	
Asphalt workers		2974 (4.5%)	
Crane operators		2935 (4.5%)	
Repairers		2077 (3.2%)	
Refrigerator technicians		801 (1.2%)	
Woodworkers			52,792 (37%)
Concrete workers			32,278 (23%)
Painters			19,368 (14%)
Ground prep workers			8991 (6.3%)
Brick layers			8772 (6.2%)
Sheet-metal worker			7940 (5.6%)
Floor layers			3462 (2.4%)
Rock blasters			3403 (2.4%)
Insulators			2009 (1.4%)
Glaziers			1847 (1.3%)
Roofers			829 (0.6%)

Data presented as *n* (%)

## Appendix 2. Unadjusted and adjusted differences in systolic blood pressure (in mmHg) between occupational physical activity (OPA) groups stratified by age

**Table:**

	Low–medium OPA	Low–high OPA	Medium–high OPA
Unadjusted			
20–29	− 0.23 (− 0.57, 0.10)	− **0.39 (**− **0.71, **− **0.07)**	− 0.16 (− 0.35, 0.03)
30–39	− **0.73 (**− **1.01, **− **0.45)**	− **0.65 (**− **0.91, **− **0.39)**	0.08 (− 0.12, 0.28)
40–49	− **1.09 (**− **1.43, **− **0.74)**	− **0.67 (**− **0.98, **− **0.36)**	**0.41 (0.16, 0.68)**
50–59	− **1.03 (**− **1.52, **− **0.54)**	− **1.59 (**− **2.01, **− **1.16)**	− **0.56 (0.92, **− **0.19)**
60–69	− 0.23 (− 1.00, 0.54)	− **0.64 (**− **1.24, **− − **0.03)**	− 0.41 (− 0.99, 0.18)
Adjusted			
20–29	− 0.20 (− 0.53, 0.13)	− **0.50 (**− **0.82, **− **0.19)**	− **0.30 (**− **0.50, **− **0.11)**
30–39	− **0.59 (**− **0.86, **− **0.31)**	− **0.75 (**− **1.00, **− **0.49)**	− 0.16 (− 0.36, 0.03)
40–49	− **0.85 (**− **1.18, **− **0.52)**	− **0.71 (**− **1.01, **− **0.40)**	0.14 (− 0.11, 0.40)
50–59	− **1.25 (**− **1.72, **− **0.78)**	− **1.49 (**− **1.90, **− **1.08)**	− 0.25 (− 0.60, 0.10)
60–69	− 0.64 (− 1.40, 0.12)	− **1.05 (**− **1.64, **− **0.45)**	− 0.40 (− 0.99, 0.17)

Unit: mmHg (95% confidence interval). Adjusted: BMI category, smoking status, and age. Numbers in bold indicate statistically significant differences, i.e. confidence intervals not containing zero

## Appendix 3. Unadjusted and adjusted differences in diastolic blood pressure (in mmHg) between occupational physical activity (OPA) groups stratified by age

**Table:**

	Low–medium OPA	Low–high OPA	Medium–high OPA
Unadjusted			
20–29	**1.06 (0.81, 1.32)**	**1.25 (1.01, 1.49)**	**0.18 (0.04, 0.33)**
30–39	**0.30 (0.09, 0.50)**	**0.67 (0.48, 0.86)**	**0.37 (0.23, 0.52)**
40–49	− 0.1 (− 0.33, 0.14)	**0.46 (0.25, 0.67)**	**0.56 (0.38, 0.73)**
50–59	0.09 (− 0.19, 0.36)	0.04 (− 0.20, 0.28)	− 0.05 (− 0.25, 0.15)
60–69	0.23 (− 0.17, 0.63)	0.05 (− 0.27, 0.36)	− 0.18 (− 0.49, 0.12)
Adjusted			
20–29	**0.88 (0.63, 1.13)**	**0.89 (0.65, 1.13)**	0.01 (− 0.13, 0.16)
30–39	**0.28 (0.08, 0.48)**	**0.47 (0.28 (0.65)**	**0.19 (0.05, 0.33)**
40–49	0.06 (− 0.16, 0.28)	**0.39 (0.19, 0.59)**	0.33 (0.16, 0.50)
50–59	− 0.05 (− 0.31, 0.21)	− 0.07 (− 0.30, 0.16)	− 0.02 (− 0.21, 0.18)
60–69	0.01 (− 0.38, 0.41)	− 0.25 (− 0.56, 0.06)	− 0.26 (− 0.56, 0.04)

Unit: mmHg (95% confidence interval). Adjusted: BMI category, smoking status, and age. Numbers in bold indicate statistically significant differences, i.e. confidence intervals not containing zero
